# Activated Microglia Disrupt the Blood-Brain Barrier and Induce Chemokines and Cytokines in a Rat *in vitro* Model

**DOI:** 10.3389/fncel.2018.00494

**Published:** 2018-12-13

**Authors:** Yukari Shigemoto-Mogami, Kazue Hoshikawa, Kaoru Sato

**Affiliations:** ^1^Laboratory of Neuropharmacology, Division of Pharmacology, National Institute of Health Sciences, Kawasaki-ku, Japan; ^2^Department of Neuropharmacology, Interdisciplinary Graduate School of Medicine, University of Yamanashi, Yamanashi, Japan

**Keywords:** BBB disruption, microglia, inflammation, cytokine, chemokine

## Abstract

Severe neuroinflammation is associated with blood brain barrier (BBB) disruption in CNS diseases. Although microglial activation and the subsequent changes in cytokine/chemokine (C/C) concentrations are thought to be key steps in the development of neuroinflammation, little data are available concerning the interaction of microglia with BBB cells. In this study, we investigated this interaction by adding LPS-activated microglia (LPS-MG) to the abluminal side of a BBB model composed of endothelial cells (EC), pericytes (Peri) and astrocytes (Ast). We then examined the abluminal concentrations of 27 C/Cs and the interactions between the LPS-MG and BBB cells. LPS-MG caused collapse of the BBB, revealed by decreases in the trans-endothelial electrical resistance (TEER) and by changes in the expression levels of tight junction (TJ) proteins. Under these conditions, 19 C/Cs were markedly increased on the abluminal side. Unexpectedly, although LPS-MG alone released 10 of the 19 C/Cs, their concentrations were much lower than those detected on the abluminal side of the BBB model supplemented with LPS-MG. Co-culture of LPS-MG with Ast caused marked increases in 12 of the 19 C/Cs, while co-culture of LPS-MG with EC and Peri resulted in a significant increase in only 1 of the 19 C/Cs (fractalkine). These results suggest that C/C dynamics in this system are not only caused by activated microglia but also are due to the interaction between activated microglia and astrocytes.

## Introduction

The blood brain barrier (BBB) acts as a protective barrier of the central nervous system (CNS) against potential neurotoxic molecules (Abbott et al., 2010; Serlin et al., 2015). BBB functions are regulated by various types of cells that belong to a neurovascular unit (NVU) (Zlokovic, 2011; Keaney and Campbell, 2015). Recent reports have clarified that microglia not only act as the CNS-resident immune cells but also have important physiological roles in the CNS (Shigemoto-Mogami et al., 2014; Sato, 2015). Microglia sense slight changes in the surrounding environment and associate along the brain capillaries (Nimmerjahn et al., 2005). In a variety of pathological conditions (Oby and Janigro, 2006; Desai et al., 2007; McCaffrey et al., 2008; Bataveljic et al., 2014; Ortiz et al., 2014; Kamphuis et al., 2015; Li et al., 2015; van de Haar et al., 2016), functional impairment of BBB has been reported (Zhao et al., 2015; Almutairi et al., 2016) and is often correlated with disease severity. Microglia adopt an activated form in pathological conditions in which BBB breakdown is a hallmark (Nakajima and Kohsaka, 1993; Kreutzberg, 1996; Graeber and Streit, 2010; Salter and Beggs, 2014; Streit et al., 2014). Furthermore, activated microglia produce inflammatory cytokines such as TNFα and IL-1β (Nishioku et al., 2010; Yang et al., 2015) that increase BBB permeability and downregulate TJ proteins (Gu et al., 2015; Almutairi et al., 2016). However, little is known about the comprehensive changes in cytokines/chemokines (C/Cs) and the interaction between microglia and the other NVU cells in neuroinflammation associated with BBB collapse. In this study, we measured the concentrations of 27 C/Cs when BBB collapse was induced by activated microglia in an *in vitro* BBB model (Nakagawa and Niwa, 2009; Nakagawa et al., 2009). We found that the interactions of activated microglia with pericytes and endothelial cells, and with astrocytes were critical in determining the final concentrations of C/Cs.

## Materials and Methods

This study was carried out in accordance with the principles of the Basel Declaration and recommendations of Guide for the Care and Use of Laboratory Animals, the Animal Research Committee of the National Institute of Health Sciences, Japan. The protocol was approved by the Animal Research Committee of the National Institute of Health Sciences, Japan.

### Materials

Bovine serum albumin (BSA), Evans blue, sodium fluorescein (NaF) and anti-β-actin antibody (A5316) were purchased from Sigma-Aldrich (St. Louis MO, United States). Fetal bovine serum (FBS) and Dulbecco's Modified Eagle's Medium (DMEM) were purchased from Life Technologies (Grand Island, NY, United States). The rat *in vitro* model of the BBB (RBT-24H) was purchased from PharmaCo-Cell Company Ltd. (Nagasaki, Japan). Anti-ZO-1 (33-9100), anti-claudin5 (35-2500), and anti-occludin (33-1500) antibodies were purchased from Invitrogen (Camarillo CA, United States). Anti-GRO KC (AF-515), anti-GFAP (AF-2594) antibodies were purchased from R&D systems (Minneapolis, MN, United States). Anti-Iba1 (019-19741) antibody and DAPI were purchased from Wako (Osaka, Japan). The MILLIPLEX MAP Rat Cytokine/Chemokine Panel was purchased from Merck Millipore (Billerica MA, United States). SuperSignal West Femto Substrate was purchased from Thermo Scientific (Rockford IL, United States). Can Get Signal was purchased from TOYOBO (Osaka, Japan).

### Preparation of the Rat *in vitro* BBB Model

**Figure 1:**
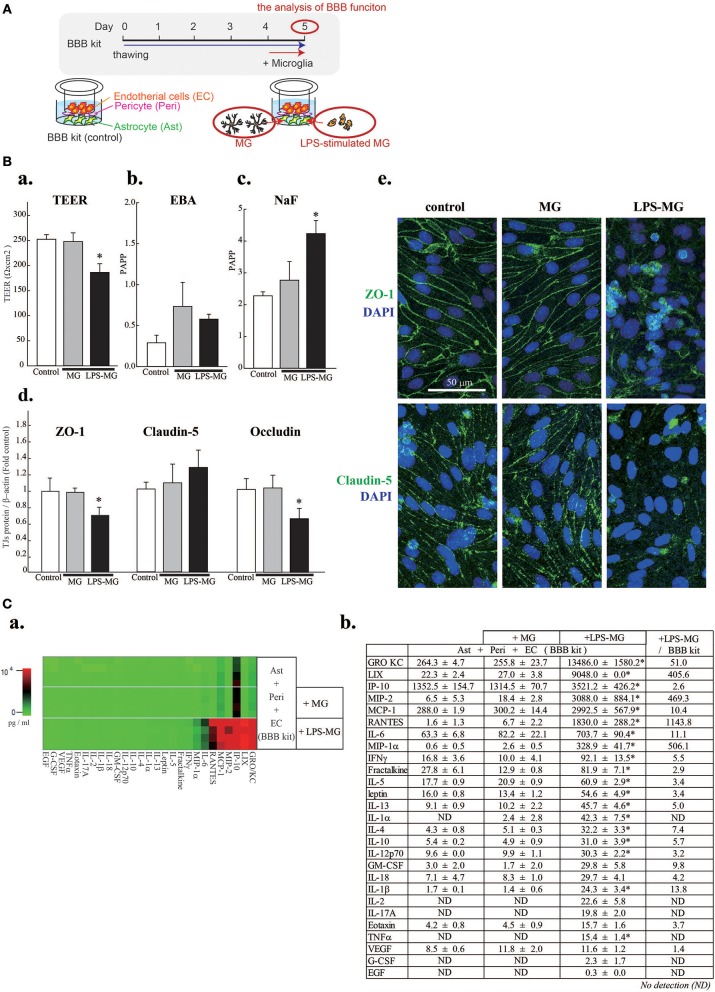
Activated microglia disrupt BBB barrier functions and cause concentration changes of 19 C/Cs. **(A)** Schematic diagram of the experiment. **(B)** Effects of LPS-MG on TEER (a), and the permeability of EBA (b) and NaF (c). Effects of LPS-MG on the expression levels of TJ proteins (d). Immunocytochemistry of ZO-1 (top) and claudin5 (bottom) (e). Scale bar indicates 50 μm. *N* = 4, ^*^ < 0.05 vs. control, ANOVA followed by Tukey's test. Error bars represent the s.e.m. **(C)** Comprehensive quantitative measurement of C/C concentrations in the medium of the abluminal side of the BBB model one day after incubation with LPS-MG. (a) Heat map of the concentrations of all 27 C/Cs. (b) Concentrations and their fold changes. The fold change represents the C/C concentration ratio of LPS-MG + BBB to BBB alone. Asterisks indicate a significant increase compared to the BBB alone. *N* = 4, ^*^ < 0.05 vs. control, ANOVA followed by Tukey's test. The reproducibility of the data was confirmed by 3 independent experiments.

The *in vitro* BBB model was cultured according to the manufacturer's protocol. Microglia were added to the abluminal side and incubated for 1 day (Figure 1A).

### Microglial Cell Culture

Rat microglia were cultured as previously described (Nakajima et al., 1992; Nakajima and Kohsaka, 1993; Shigemoto-Mogami et al., 2014). To activate microglia, they were incubated with 1 μg/ml LPS for 1 h (LPS-MG), which has already been shown to induce inflammatory reaction in our preliminary experiments and another's report (Huang et al., 2018). Microglia were then washed twice, and transferred to the abluminal side of the BBB model at 5.0 × 10^4^ cells/cm^2^.

### Measurement of the Transendothelial Electrical Resistance (TEER)

The TEER was measured by an Endohm resistance meter (World Precision Instruments, FL. United States).

### Measurement of Transcellular Transport and Paracellular Transport

To measure transcellular transport and paracellular transport, we measured the permeabilities of Evans blue albumin (EBA) and NaF, respectively. EBA (165 μg/ml) and NaF (10 μg/ml) were added to the luminal side. PBS-H (10 mM HEPES, 25 mM glucose in PBS) was added to the transmembranes (30 min) and the concentrations of NaF and EBA in the PBS-H were measured using a Spectra Max (Molecular Devices, CA. United States). The permeability coefficient (P_app_) was calculated using the following formula: P_app_ (cm/s) = V_A_/A x [C]_luminal_ x Δ [C]_Abluminal_/Δt.

### Western Blotting of TJ Proteins

The transmembranes with EC and Peri were lysed with sample buffer (62.5 mM Tris, 2% SDS, 10% glycerin, 0.0125% bromophenol blue, pH 6.8), and homogenized on ice. The concentrations of primary antibodies: anti-ZO-1 [1:1,000], anti-claudin5 [1:2,000], anti-occludin [1:1,000], anti-β-actin [1:5,000]. We have confirmed that the bands were detected with their specific molecular weights, i.e., ZO-1(225 kDa), claudin5 (24 kDa), occludin (65 kDa), and β-actin (42 kDa). The level of β-actin was not changed in any experimental conditions.

### Immunocytochemistry

The expressions of ZO-1 and claudin5 in EC and Peri, and the expression of GRO KC in co-culture of Ast and MG were examined immunocytochemically (Shigemoto-Mogami et al., 2014). The primary antibodies were used in the following concentrations: anti-ZO-1 [1:100], anti-claudin5 [1:100], anti-GRO KC [1:100], anti-GFAP [1:50], anti-Iba1 [1:100]. For visualization of nuclei, DAPI was used [1:1,000].

### Measurement of C/Cs

The concentrations of 27 rat C/Cs (IL-1α, IL-1β, IL-2, IL-4, IL-5, IL-6, IL-10, IL-12 (p70), IL-13, IL-17, IL-18, EGF, eotaxin, fractalkine, G-CSF, GM-CSF, GRO/KC, IFN-γ, IP-10, leptin, LIX, MCP-1, MIP-1α, MIP-2, RANTES, TNFα, and VEGF) were measured by MILLIPLEX MAP Multiplex Immunoassay Kits (Rat Cytokine/Chemokine Magnetic bead kit RCYPMX27-MAG).

### Data Analysis and Statistics

The statistical analysis was performed using ANOVA followed by Tukey's test. Differences were considered significant at a value of *p* < 0.05. The reproducibility of the data was confirmed by 3 independent experiments.

## Results

We first attempted to reproduce the BBB disruption observed in neuroinflammation by adding LPS-MG to the abluminal side of the BBB model (Figures 1A,Ba–e). A 1 day incubation with LPS-MG significantly decreased the TEER and increased paracellular transport (NaF). In addition, the expression levels of occludin and ZO-1 were significantly decreased (Figure 1Bd). As revealed by immunostaining of ZO1 and claudin5, TJ structures were collapsed by LPS-MG (Figure 1Be). When we added microglia without LPS stimulation, no changes were detected in these parameters. We then measured the concentrations of 27 C/Cs on the abluminal side of the BBB model. The basal levels of C/Cs, without microglia, were identical to levels after adding unstimulated microglia concentrations (shown in a heat map, Figure 1Ca and quantitatively, Figure 1Cb). However, adding LPS-MG significantly increased the concentrations of 19 C/Cs (GRO/KC, LIX, IP-10, MIP-2, MCP-1, RANTES, IL-6, MIP-1α, IFN-γ, fractalkine, IL-5, leptin, IL-13, IL-1α, IL-4, IL-10, IL-12 (p70), IL-1β, and TNFα). The right column of Figure 1Cb indicates the ratios of C/Cs with LPS-MG to those at basal levels, i.e., the induction strength.

**Figure 2:**
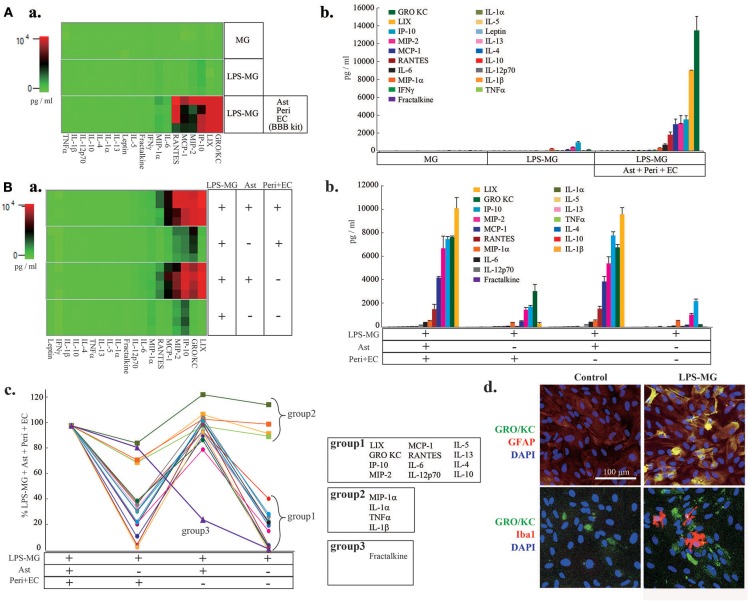
The interaction of LPS-MG with the other NVU cells is important for C/C dynamics. **(A)** After 1 day of incubation with LPS-MG, the concentrations of all 27 C/Cs were measured. (a) The heat map of the 27 C/Cs in microglia alone, LPS-MG alone and the LPS-MG + BBB model. (b) Comparison of the concentrations of the 19 C/Cs upregulated by LPS-MG (see Fig. 1) in the above three culture conditions. **(B)** The 19 C/C concentrations in the LPS-MG + BBB model, the LPS-MG + Peri + EC, LPS-MG + Ast, and LPS-MG. (a) Heat map of the 19 C/C concentrations in the above four culture conditions. (b,c) Concentrations of the C/Cs in all the experimental conditions: (b) raw concentrations; (c) those normalized to the LPS-MG + BBB model. The 19 C/Cs were divided into three groups. (d) Typical expression pattern of cell type specific markers (GFAP, Iba1) (red) and that of GRO KO (green) in the co-culture of LPS-MG and astrocytes were shown. Scale bar indicates 100 μm. The reproducibility of the data was confirmed by 3 independent experiments.

We next attempted to identify the cell types responsible for the changes in C/Cs (Figure 2). We first measured C/C concentrations in supernatants of microglia alone, of LPS-MG alone, and of the medium on the abluminal side of the BBB model supplemented with LPS-MG (Figure 2Aa,b). Microglia alone did not affect the concentrations of C/Cs, but LPS-MG increased 10 C/Cs (IL-1β, IL-10, IL-12 (p70), GRO/KC, IP-10, MCP-1, MIP-1α, RANTES, MIP-2, and TNFα). However, the concentrations of these C/Cs were much lower than C/Cs of the BBB model supplemented with LPS-MG. These results suggest that the interaction of LPS-MG with BBB cells is important in determining the final concentrations of C/Cs. To determine which of the BBB components increased the C/C concentrations, we compared the C/C concentrations in the entire LPS-MG + BBB model to that of LPS-MG co-cultured only with Peri + EC, co-cultured only with Ast, and LPS-MG cells alone, without any BBB components (Figure 2B). The heat map of C/Cs in the LPS-MG + Ast and LPS-MG + BBB conditions were very similar (Figure 2Ba). Figure 2Bb and c show the concentrations of the respective C/Cs in the four culture conditions. The 19 C/Cs appeared to be divided into three groups. Group 1 (LIX, GRO/KC, IP-10, MIP-2, MCP-1, RANTES, IL-6, IL-12 (p70), IL-5, IL-13, IL-4, and IL-10) included C/Cs of which the final concentrations were determined by the interaction between LPS-MG and Ast. When we performed double immunostaining of GRO KC, one of the members of this group, along with cell type specific markers, we found GRO KC associated with astrocytes after the stimulation with LPS-MG (Figure 2Bd, top), but not with microglia (bottom). Group 2 (MIP-1α, IL-1α, TNFα, and IL-1β) included C/Cs of which the final concentrations depended on LPS-MG. Group 3 included fractalkine; the concentration was mainly determined by the Peri + EC condition, and Ast suppressed its release.

## Discussion

The addition of activated microglia to the abluminal side of the BBB model caused a collapse of the BBB. For example, the membrane-specific localization of ZO-1 and claudin5 almost disappeared after microglial addition. Activated microglia also induced drastic changes in the concentrations of C/Cs, indicating that microglial activation can trigger BBB disruption during neuroinflammation. Among 19 C/Cs that showed significant changes in our experiment, IP-10 (Chai et al., 2015), MCP-1 (Yao and Tsirka, 2014), IL-6 (Paul et al., 2003), IL-1β (Argaw et al., 2006; Wang et al., 2014), and TNFα (Afonso et al., 2007) have previously been reported to induce BBB disruption. The concentrations of GRO/KC (Maysami et al., 2015), LIX (Wang et al., 2016), MIP-2 (Shaftel et al., 2007), RANTES (Ubogu et al., 2006), and MIP-1α (Maysami et al., 2015) are reported to be elevated at the time of BBB collapse, which causes infiltration of monocytes and T cells into the CNS, thereby worsening BBB disruption. The C/Cs described above may act as both a cause and a consequence of the neuroinflammation.

Many studies have suggested that activated microglia themselves disrupt BBB integrity by releasing inflammatory C/Cs (Yenari et al., 2006; da Fonseca et al., 2014). However, we found that although activated microglia alone induced significant elevations of GRO/KC, IP-10, MIP-2, MCP-1, RANTES, MIP-1α, IL-12 (p70), IL-10, IL-1β, TNFα, and IL-1α, their concentration changes were slight. The concentrations of 12 C/Cs (LIX, GRO/KC, IP-10, MIP-2, MCP-1, RANTES, IL-6, IL-12 (p70), IL-5, IL-13, IL-4, and IL-10) were mainly determined by the interaction between astrocytes and LPS-MG. The 7 most highly concentrated C/Cs (GRO KC, LIX, IP-10, MIP-2, MCP-1, RANTES, and IL-6) in Figure 1Bb were included in this group. LIX (Wang et al., 2016), GRO/KC (Maysami et al., 2015), IP-10 (Chai et al., 2015), MIP-2 (Shaftel et al., 2007), MCP-1 (Yao and Tsirka, 2014), RANTES (Ubogu et al., 2006), IL-6 (Paul et al., 2003), and IL-10 (Lin et al., 2018) play roles in BBB disruption during neuroinflammation. By immunostaining, we confirmed that the main resource of GRO KC was astrocytes, while only minor signals were detected in LPS-MG. Further experiments are necessary to clarify the contribution of astrocytes and activated microglia to the final concentration of each cytokine. The concentrations of IL-1β, TNFα, IL-1α, and MIP-1α in the above 4 culture conditions were the same as those with LPS-MG alone, indicating that activated microglia are the predominant source of these C/Cs. However, their concentrations were lower than those in the co-culture of LPS-MG and Ast. These four C/Cs are well-known pro-inflammatory C/Cs (Wang et al., 2014; Rochfort et al., 2016) and might trigger the subsequent serial C/C concentration changes leading to BBB collapse. The concentration change of fractalkine was unique. The main source of fractalkine was Peri + EC, i.e., blood vessels, and microglia suppressed the fractalkine release.

In this study, activated microglia-induced C/Cs were categorized into three groups based on the main sources, i.e., microglia, Ast, and EC + Peri (blood vessels). Of note, microglia-triggered elevation of Ast C/Cs was remarkable in terms of both quantity and variation. BBB collapse and the subsequent infiltration of peripheral immune cells into brain parenchyma drastically worsen neuroinflammation (Dos Passos et al., 2016; Zenaro et al., 2017). It therefore seems likely that signals between microglia and Ast are potent therapeutic targets for neuroinflammation.

## Author Contributions

KS: designed research; YS-M, KH, and KS: performed research; YS-M, KH, and KS: analyzed data; YS-M and KS: wrote the paper.

### Conflict of Interest Statement

The authors declare that the research was conducted in the absence of any commercial or financial relationships that could be construed as a potential conflict of interest.
